# Orientation Dependence of Elastic and Piezoelectric Properties in Rhombohedral BiFeO_3_

**DOI:** 10.3390/ma11122441

**Published:** 2018-12-02

**Authors:** Gang Jian, Fei Xue, Yuhang Guo, Chao Yan

**Affiliations:** 1 School of Materials Science and Engineering, Jiangsu University of Science and Technology, Zhenjiang 212003, China; guoyuhang@just.edu.cn; 2 School of Materials Science and Engineering, Georgia Institute of Technology, Atlanta, GA 30332, USA; 3 Center of Collaboration and Innovation, Jiangxi University of Technology, Nanchang 330098, China; xuefei_work@126.com

**Keywords:** bismuth ferrite, orientation dependence, coordinate transformation, elasticity, piezoelectricity

## Abstract

Through a coordinate transformation approach, crystal orientation dependences of elastic and piezoelectric properties at room temperature have been investigated in a three-dimensional space for rhombohedral bismuth ferrite (BiFeO_3_). Elastic constants (stiffnesses) *c*_11′_, *c*_12′_, c_13′_ and piezoelectric constants *d*_15′_, *d*_31′_, *d*_33′_ along arbitrary orientations were obtained based on crystalline asymmetry characteristics of *3m* point group BiFeO_3_. Parameters along specific orientations obtaining the largest values were presented. The *max* *c*_11′_ = 213 × 10^9^ N/m^2^ could be achieved in planes with ϕ = 0° and 90°. The *max* *c*_12′_ = *c*_13′_ = 132.2 × 10^9^ N/m^2^ could be achieved along directions at *θ* = 13° and *θ* = 77° inside three mirror planes, respectively. The *max* *d*_15′_ = 27.6 × 10^−^^12^ C/N and the *max* *d*_31′_ = 12.67 × 10^−^^12^ C/N could be both obtained along directions at *θ* = 69° inside mirror planes. The *max* *d*_33′_ = 18 × 10^−^^12^ C/N could be obtained at *θ* = 0°, along the spontaneous polarization axis. By adopting optimal directions, the elastic and piezoelectric parameters of BiFeO_3_ could be significantly enhanced which shows applications for the growth of BeFeO_3_ films with preferred orientations and enhanced properties.

## 1. Introduction

Bismuth ferrite (BiFeO_3_) was first actively pursued as a room temperature single-phase multiferroic material for its coexistence of magnetic order and electric order, high Curie point, and G-type antiferromagnetic Neel point [1,2,3,4]. Then, as BiFeO_3_ exhibits good ferroelectric and piezoelectric properties, it was also studied as a piezoelectric phase to combine other magnetostrictive phase to form composite multiferric with tailored properties, such as BiFeO_3_-CoFe_2_O_4_ [5], BiFeO_3_-CuFe_2_O_4_ [6], etc., which shows potential technical applications in multi-state magnetoelectric memories [7], weak magnetic fields detectors [8], and other novel sensors [9,10]. As a lead-free piezoelectric material, BiFeO_3_ could be highly applied in piezoelectric MEMS devices because of the spontaneous polarization of as high as 100 μC/cm^2^ [11] and the high Curie temperature [3]. Besides, BiFeO_3_ thin films are reported to have lower dielectric constants than Pb-based piezoelectric materials which could generate high electromechanical coupling abilities [12,13]. On raising piezoelectric constants of BiFeO_3_, previous studies were focused on doping transition metals such as Sm, Yb, Ho in A site and Sc, In in B site of BiFeO_3_, piezoelectric constants d_33_ of up to ~20–~50 pC/N were achieved [14,15]. Most piezoelectrics have asymmetrical crystal structures, which result high anisotropy of parameters of the materials. Piezoelectric thin films with preferred orientations can obtaion enhanced or decreased properties compared with grain random distributed materials [16,17]. This also applies to doped BiFeO_3_ with unchanged crystal structures. Whether the properties will be enhanced or decreased is strongly determined by asymmetry characteristics of crystal, i.e., the point groups. The crystal structure of BiFeO_3_ below 1100 K belongs to a rhombohedral system with point group of *3m* [18]. It is meaningful to have a precise description of properties-orientation relations to guide further experimental works. In this research, using the coordinate transformation method, we investigated the orientation dependences of elastic and piezoelectric parameters of rhombohedral BiFeO_3_ with the *3m* point group. Precise relations between values of parameters and arbitrary orientations were given, and orientations along which the maximum and minimum of these parameters could be obtained were specified. The result shows applications for the growth of BiFeO_3_ films with an oriented structure and enhanced properties.

## 2. Methods 

**Figure 1:**
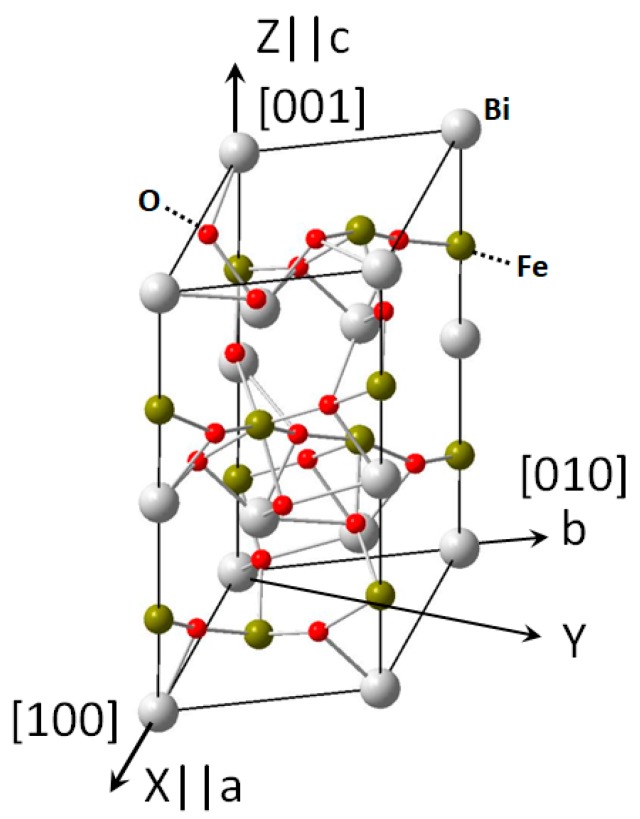
Illustration of the crystal structure of BiFeO_3_ (*R3C* space group, *3m* point group). Lattice constants: *a* = *b* = 0.56 nm, *c* = 1.395 nm; *α* = *β* = 90°, *γ* = 120°. The chosen crystallographic coordinate system: *X*||*a*, *Z*||*c*, *Y*⊥*a*. The angle between *Y* and *b*-axis is 30°.

The Curie point and the antiferromagnetic Neel point of BiFeO_3_ are 1100 [3] and 640 K [4], respectively. Below the Curie point, BiFeO_3_ is a member of rhombohedral crystal system with point group of *3m* [18]. For this type of crystal, the asymmetrical characteristics lie in such a way that it has a threefold rotation axis along the *c*-axis (also the spontaneous polarization axis, displacements of Bi relative to O in rhombohedral BiFeO_3_ [19,20], also can be denoted as [111]-axis using coordinate with oxygen octahedra in perovskite Bi framework) and three mirror planes which are 120° apart (two of them are parallel to *a*- and *b*-axes, respectively). Because of *α* = *β* = 90° and *γ* = 120° for rhombohedral BiFeO_3_ crystal, the physical orthogonal coordinate system does not coincide rightly with *a*-, *b*-, and *c*-axes. The chosen coordinate system in this study is as follows: *X*-axis and *Z*-axis are chosen along *a*-axis and *c*-axis (*i.e.*, *X*||*a*, *Z||c*), respectively; *Y*-axis is determined by using the right-hand rule, which is perpendicular to *a*-axis and at a 30° angle with *b*-axis (Figure 1).

To determine the anisotropy of properties originating from asymmetry of crystals, we use the coordinate system transformation method that is related to transforming the property tensors to obtain values in any arbitrary direction. It contains two-step rotations to reach a desired direction from the original coordinate system. Firstly, rotation through a clockwise angle ϕ about the *Z*-axis, the original orthogonal coordinates *XYZ* can be changed into a set of new coordinates *X’Y’Z’* (*Z’*||*Z*); secondly, rotation through a clockwise angle *θ* about the *X’*-axis; the coordinate system *X’Y’Z’* can be changed into another set of coordinates *X”Y”Z”* (*X”*||*X’*). The transformation matrices *A_Z_* and *A_X_* corresponding to the first and the second rotations are

(1) $$A_{Z}=\left(\begin{array}{ccc} \cos\phi & \sin\phi & 0\\ -\sin\phi & \cos\phi & 0\\ 0 & 0 & 1 \end{array}\right)$$

(2) $$A_{X}=\left(\begin{array}{ccc} 1 & 0 & 0\\ 0 & \cos\theta & \sin\theta \\ 0 & -\sin\theta & \cos\theta \end{array}\right)$$

*N* and *M* are [6 × 6] bond strain transformation matrices. *a_ij_* represents the element in row *i* and column *j* in transformation matrices *A*, the matrices *N* and *M* are

(3) $$N=\left(\begin{array}{cccccc} a_{11}^{2} & a_{12}^{2} & a_{13}^{2} & a_{12}a_{13} & a_{13}a_{11} & a_{11}a_{12}\\ a_{21}^{2} & a_{22}^{2} & a_{23}^{2} & a_{22}a_{23} & a_{23}a_{21} & a_{21}a_{22}\\ a_{31}^{2} & a_{32}^{2} & a_{33}^{2} & a_{32}a_{33} & a_{33}a_{31} & a_{31}a_{32}\\ 2a_{21}a_{31} & 2a_{22}a_{32} & 2a_{23}a_{33} & a_{22}a_{33}+a_{32}a_{23} & a_{23}a_{31}+a_{33}a_{21} & a_{21}a_{32}+a_{31}a_{22}\\ 2a_{31}a_{11} & 2a_{32}a_{12} & 2a_{33}a_{13} & a_{32}a_{13}+a_{12}a_{33} & a_{33}a_{11}+a_{13}a_{31} & a_{31}a_{12}+a_{11}a_{32}\\ 2a_{11}a_{21} & 2a_{12}a_{22} & 2a_{13}a_{23} & a_{12}a_{23}+a_{22}a_{13} & a_{13}a_{21}+a_{23}a_{11} & a_{11}a_{22}+a_{21}a_{12} \end{array}\right)$$

(4) $$M=\left(\begin{array}{cccccc} a_{11}^{2} & a_{12}^{2} & a_{13}^{2} & 2a_{12}a_{13} & 2a_{11}a_{13} & 2a_{11}a_{12}\\ a_{21}^{2} & a_{22}^{2} & a_{23}^{2} & 2a_{22}a_{23} & 2a_{21}a_{23} & 2a_{21}a_{22}\\ a_{31}^{2} & a_{32}^{2} & a_{33}^{2} & 2a_{32}a_{33} & 2a_{31}a_{33} & 2a_{31}a_{32}\\ a_{21}a_{31} & a_{22}a_{32} & a_{23}a_{33} & a_{22}a_{33}+a_{32}a_{23} & a_{23}a_{31}+a_{33}a_{21} & a_{21}a_{32}+a_{31}a_{22}\\ a_{11}a_{31} & a_{12}a_{32} & a_{13}a_{33} & a_{32}a_{13}+a_{12}a_{33} & a_{33}a_{11}+a_{13}a_{31} & a_{31}a_{12}+a_{11}a_{32}\\ a_{11}a_{21} & a_{12}a_{22} & a_{13}a_{23} & a_{12}a_{23}+a_{22}a_{13} & a_{13}a_{21}+a_{23}a_{11} & a_{11}a_{22}+a_{21}a_{12} \end{array}\right)$$

The elastic constants, including the compliance *s_ij_* (*i, j* = 1–6) (m^2^/N) and the stiffness *c_ij_* (*i, j* = 1–6) (N/m^2^), are defined by Hooke’s Law containing the relations between the strain *x* and the stress *X*, (*x*) = (*s*) (*X*) and (*X*) = (*c*) (*x*). The piezoelectric coefficient *d_ij_* (*i* = 1–3, *j* = 1–6) (C/N), relates polarization *P*, to stress *X* in the relation of (*P*) = (*d*) (*X*). For a three-dimensional space coordinate system, all physical quantities are presented in matrix forms. Matrices of elastic stiffnesses *c_ij_* and piezoelectric constants *d_ij_* are presented in [6 × 6] and [3 × 6] rank tensors, respectively. According to Euler’s rotation laws, physical quantity matrices in a new orientation are obtained by two-step product operations with transformation matrices *A*, *N*, and *M* [21,22].

(5) $$\left(c{}^{\prime }\right)=M_{X}\cdot M_{Z}\cdot \left(c\right)\cdot M_{Z}^{t}\cdot M_{X}^{t}$$

(6) $$\left(d{}^{\prime }\right)=A_{X}\cdot A_{Z}\cdot \left(d\right)\cdot N_{Z}^{t}\cdot N_{X}^{t}$$

The symbols ‘′’ and ‘*t*’ represent parameters in the new coordinate system and transpose matrix, respectively. The key to calculate Equations (5) and (6) is using the right original matrices (at *θ* = ϕ = 0°) which are obtained from Neumann’s principle changing with different point groups. The original matrices of (*c*) and (*d*) for *3m* point group can be obtained from Ref. [20]. The elastic stiffnesses *c_ij_* of BiFeO_3_ at room temperature were obtained from Refs. [23,24]. The piezoelectric constants *d_ij_* of BiFeO_3_ at room temperature were obtained from Refs. [24,25]. Three-dimensional and two-dimensional representation graphs of parameters were drawn using Maple 18, a mathematical software program.

## 3. Results and Discussion

**Figure 2:**
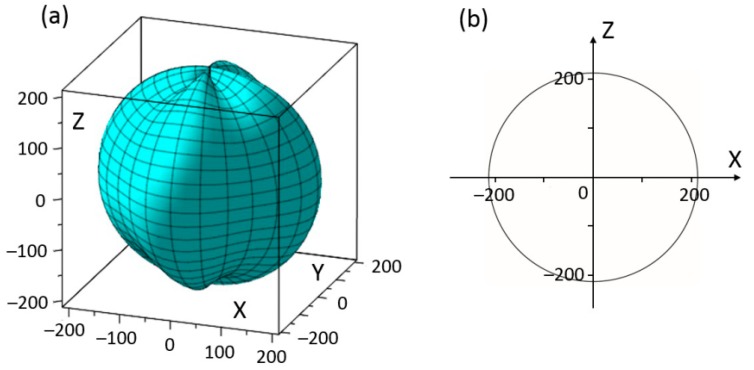
(**a**) Schematic diagram of elastic stiffness *c*_11_ by the experimental data of BiFeO_3_; (**b**) elastic stiffness *c*_11_ of BiFeO_3_ in *XOZ* plane (also applies to *YOZ* plane, unit GPa).

In both three-dimensional and two-dimensional graphs, the distance between a point and the original point represents the absolute value of the relative physical quantity in the relative direction. Orientational dependences of elastic constants *c_ij_*_′_ were obtained by calculating (*c*′) in Equation (5) using data of five independent elastic constants (*c*_11_, *c*_12_, *c*_13_, *c*_33_, and *c*_44_) for rhombohedral BiFeO_3_. Figure 2a shows the three-dimensional representation of the first term elastic constant *c*_11′_. In the geometry in Figure 2a, moderate changes of *c*_11′_ could be observed with rhombohedral BiFeO_3_ crystal rotating to different (*θ*, *ϕ*). The *max c*_11′_ = 213 × 10^9^ N/m^2^ is along ϕ = 0° and 90° (also see Figure 2b); while the *min c*_11′_ = 188 × 10^9^ N/m^2^ is along ϕ = 45° and 135° with a 11.7% decrease from the maximum. It also indicates that along principle axes of rhombohedral BiFeO_3_, the max *c*_11′_ could be obtained.

**Figure 3:**
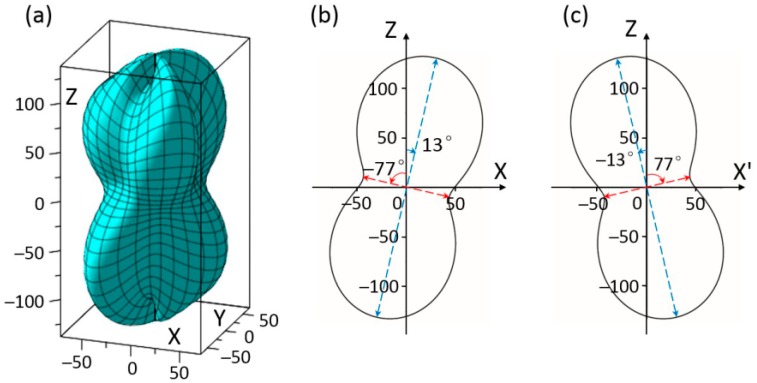
(**a**) Schematic diagram of elastic stiffness *c*_1__2_ by the experimental data of BiFeO_3_; (**b**) elastic stiffness *c*_1__2_ of BiFeO_3_ in *XOZ* plane at ϕ = 0°; (**c**) elastic stiffness *c*_1__2_ of BiFeO_3_ in *X*’*OZ* plane at ϕ = 60° (unit GPa).

Figure 3a shows the three-dimensional representation of the second term elastic stiffness *c*_12′_ of rhombohedral BiFeO_3_. The geometry exhibits rotation symmetry in that by rotating through the *Z*-axis about 120° it coincides with each other, and by rotating through the *Z*-axis about 60° the rotated and original shapes are axisymmetric about the *Z*-axis. This is because to the symmetry elements of rhombohedral BiFeO_3_ are three mirror-planes 120° apart from each other. Figure 3b shows the cross-section plots of *c*_12′_ in *XOZ* plane at ϕ = 0° or 120°. Plot in Figure 3c at ϕ = 60° is axisymmetric with the plot of Figure 3b about the *Z*-axis. The *max c*_12′_ = 132.3 × 10^9^ N/m^2^ can be obtained at *θ* = 13° when ϕ = 0° and 120°, *θ* = −13° when ϕ = 60°. This indicates the three maximums exist in the mirror plane of rhombohedral BiFeO_3_, but they are at an angle to the spontaneous polarization axis. The *min c*_12′_ = 49 × 10^9^ N/m^2^ can be obtained at *θ* = −77°.

**Figure 4:**
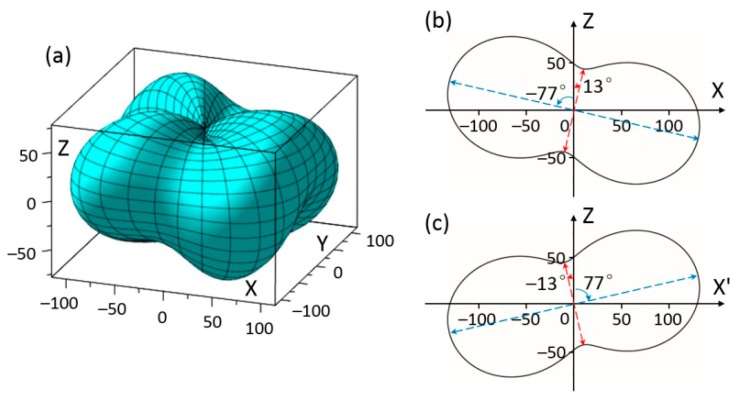
(**a**) Schematic diagram of elastic stiffness *c*_1__3_ by the experimental data of BiFeO_3_; (**b**) elastic stiffness *c*_1__3_ of BiFeO_3_ in *XOZ* plane at ϕ = 0°; (**c**) elastic stiffness *c*_1__3_ of BiFeO_3_ in *X*’*OZ* plane at ϕ = 60° (unit GPa).

Figure 4a shows the three-dimensional representation of the second term elastic stiffness *c*_13′_ of rhombohedral BiFeO_3_. Unlike *c*_12′_, large *c*_13′_ is found along the transverse directions, rather than the longitudinal directions. Symmetrical lobes of *c*_13′_ also are along mirror planes, as shown in Figure 4b,c. The *max c*_13′_ = 132 × 10^9^ N/m^2^ can be obtained at *θ* of 77° inside mirror planes. The *min c*_13′_ = 44.6 × 10^9^ N/m^2^ can be obtained at *θ* of –13°. Values of *c*_11′_, *c*_12′_, and *c*_13__′_ of rhombohedral BiFeO_3_ along several chosen orientations are shown in Table 1.

**Figure 5:**
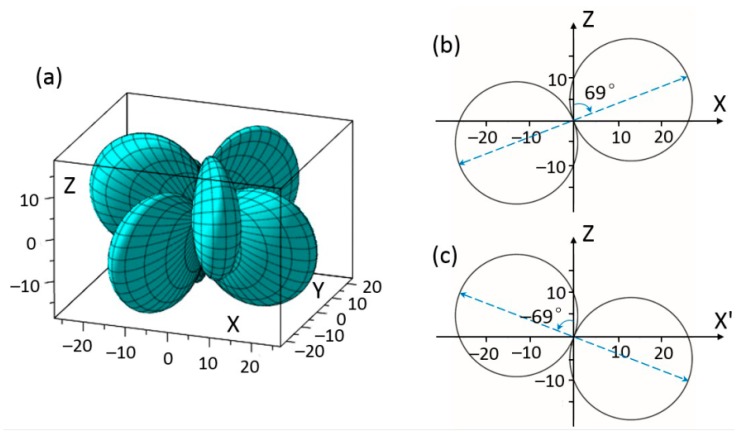
(**a**) Schematic diagram of piezoelectric constant *d*_1__5_ by the experimental data of BiFeO_3_; (**b**) piezoelectric constant *d*_1__5_ of BiFeO_3_ in *XOZ* plane at ϕ = 0°; (**c**) piezoelectric constant *d*_1__5_ of BiFeO_3_ in *X*’*OZ* plane at ϕ = 60° (unit 10^−^^12^ C/N).

Orientational dependences of piezoelectric constants *d_ij_*_′_ were obtained by calculating (*d*′) in Equation (6) using data of four independent elastic constants (*d*_15_, *d*_22_, *d*_31_, and *d*_33_) for rhombohedral BiFeO_3_. Figure 5a shows the three-dimensional representation of shear piezoelectric constant *d*_15′_. There are three pairs of lobes in the geometry shape of *d*_15′_. Each pairs are along mirror planes of *3m* BiFeO_3_, and two components of each pairs are centrosymmetric to the origin point. *d*_15′_ with large values could be obtained along orientations inside there lobes and it tends to be zero outside these lobes showing very evident spatial anisotropy. Figure 5b shows a cross-sectional plot of piezoelectric constant *d*_15′_ at ϕ = 0°, 120°, and 240° (Figure 5c is the relative plot at ϕ = 60°). The *max d*_15′_ = 27.6 × 10^−12^ C/N could be obtained at *θ* = 69° inside mirror planes.

**Figure 6:**
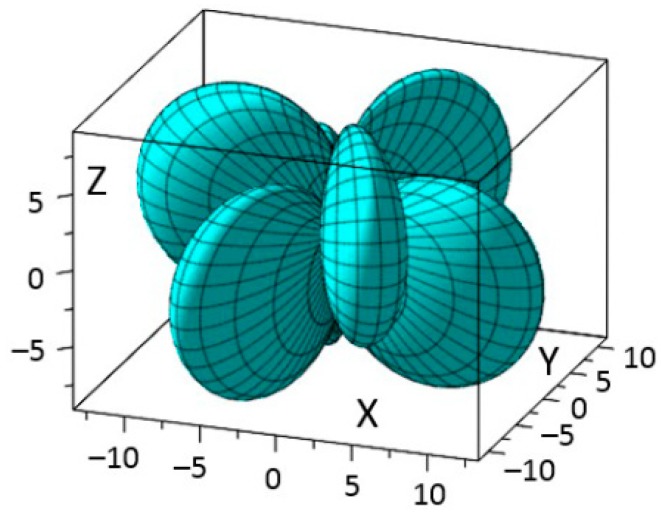
Schematic diagram of piezoelectric constant *d*_31_ by the experimental data of BiFeO_3_ (unit 10^−^^12^ C/N).

The transverse piezoelectric constant *d*_31′_ of rhombohedral BiFeO_3_ exhibits characteristics quite similar to that of *d*_31′_, shown in Figure 6. The *max d*_31′_ = 12.67 × 10^−12^ C/N could also be obtained at *θ* = 69° inside mirror planes, which is about three times larger than *d*_31__′_ = 4.5 × 10^−12^ C/N along the [001]-axis. Notably, the transverse piezoelectric constant *d*_31′_ tends to be small along the spontaneous polarization axis ([001]-axis in this study). The result is in accordance with transverse piezoelectric stress constant values *e*_31_ (unit C/m^2^, *e* = *d*·*c*, where *d* and *c* are piezoelectric constant and elastic stiffness, respectively) of epitaxial BiFeO_3_ films with various preferred orientations, *e*_31_ along the spontaneous polarization axis and at a tilt 54.7° were −1.3 and −3.5 C/m^2^, respectively, which is also about three times larger [26].

**Figure 7:**
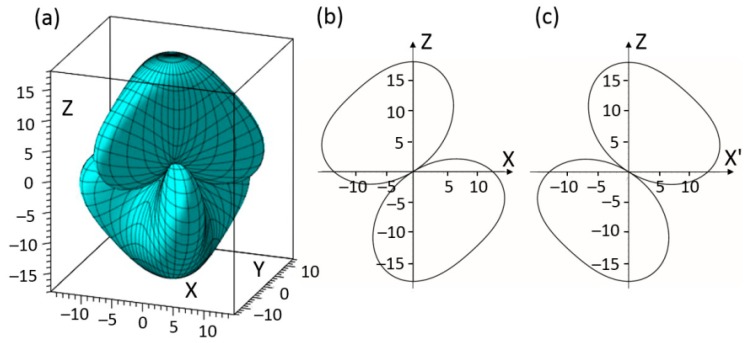
(**a**) Schematic diagram of piezoelectric constant *d*_33_ by the experimental data of BiFeO_3_; (**b**) piezoelectric constant *d*_33_ of BiFeO_3_ in *XOZ* plane at ϕ = 0°; (**c**) piezoelectric constant *d*_33_ of BiFeO_3_ in *X*’*OZ* plane at ϕ = 60° (unit 10^−^^12^ C/N).

Figure 7a shows the three-dimensional representation of longitudinal piezoelectric constant *d*_33′_ of rhombohedral BiFeO_3_. Symmetrical lobes of *d*_33′_ also can be found along mirror planes. The *max d*_33′_ = 18 × 10^−12^ C/N could be obtained at *θ* = 0° along the spontaneous polarization axis. The cross-sectional two-dimensional plots in *X’OZ* planes are shown in Figure 7b,c. The value of *d*_33′_ along [100] and [010] is 13 × 10^−12^ C/N. The result in this study is quite in accordance with *d*_33_ value of BiFeO_3_ crystal measured by Raman scattering, which is *d*_33_ = 16 × 10^−12^ C/N [27]. For epitaxial BiFeO_3_ films, because of another measurement method applied (i.e., piezoelectric force microscopy, PFM) and strain from the substrates, values of *d*_33_ of epitaxial BiFeO_3_ films tend to be larger, in a range of 20–60 × 10^−12^ C/N. However, experimental results showed epitaxial BiFeO_3_ films with oriented structure has about four times the enhancement of longitudinal piezoelectric constant compared with polycrystalline BiFeO_3_ films [28,29], which are generally in line with our results. Values of *d*_15′_, *d*_31′_, and *d*_33′_ of rhombohedral BiFeO_3_ along several chosen orientations are summarized in Table 1.

**Figure 8:**
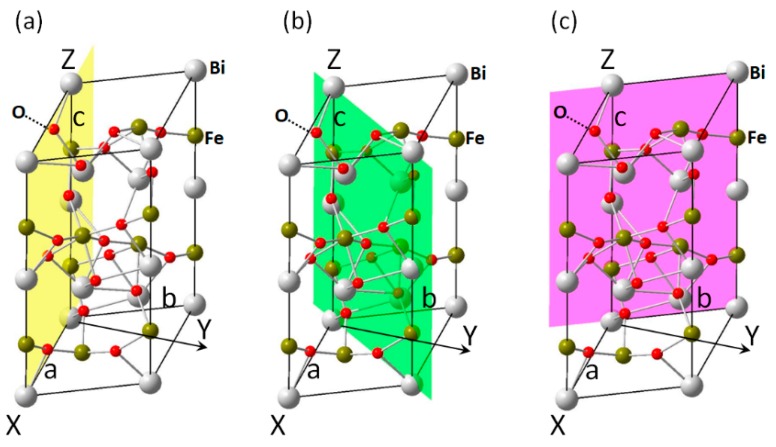
Crystalline planes of BiFeO_3_ at various rotation angles: (**a**) *ϕ* = 0°, along *a*- and *c*-axes, Miller index (010)-plane; (**b**) *ϕ* = 60°, along *c*-axis, Miller index (1(-)10)-plane; (**c**) *ϕ* = 120°, along *b*- and *c*-axes, Miller index (100)-plane. The three planes are also the three mirror symmetry planes.

From the orientation dependences of elastic and piezoelectric parameters investigated in this study, it can be found that the spatial anisotropy of *c_ij_* and *d_ij_* are related closely with the asymmetrical characteristics of rhombohedral BiFeO_3_ crystal with the *3m* point group. As there is a rotation axis that is also the *c*-axis, several parameters tend to produce extreme values along this direction: *c*_11_ and *d*_33_ have the max values while *d*_15_ and *d*_31_ have the min values. Also, as there are three mirror planes locate at ϕ = 0°, 60° (240°), and 120°, respectively (see Figure 8a–c, also denoting them with Miller index of (010), ($\bar{1}10$), and (100), respectively), the main lobes of several parameters are along the mirror planes: *c*_12_, *c*_13_, and all piezoelectric constants investigated. It also should be noted that Maxima directions are at a certain angle with the main crystalline axes, which may be attributed to the intrinsic lattice effects, which also appear in other piezoelectrics like BaTiO_3_ [30] and LiNbO_3_ [31]. The values of these parameters along arbitrary orientations were given in this study (some are listed in Table 1), which provides precise predictions for values of parameters with changing orientations. Table 1 also indicates that as a lead-free piezoelectric material, BiFeO_3_ has smaller elastic stiffnesses (larger elastic compliances) than tetragonal BaTiO_3_, and it has piezoelectric constants in the same level with those of KNbO_3_ [32,33]. Furthermore, the largest values of these parameters were given in this study, which shows applications for the growth of BiFeO_3_ films with oriented structure and enhanced properties.

## 4. Conclusions

Using the coordinate transformation method, the elastic and piezoelectric parameters at room temperature of rhombohedral BiFeO_3_ with the *3m* point group along arbitrary orientations have been investigated. Several conclusions were obtained through this study:

(1) The *max* elastic stiffness *c*_11′_ = 213 × 10^9^ N/m^2^ lies in planes with ϕ = 0° and 90°. The *max* elastic stiffness *c*_12′_ = *c*_13′_ = 132.2 × 10^9^ N/m^2^ lie in directions at *θ* = 13° and *θ* = 77° inside three mirror planes, respectively.

(2) The *max* piezoelectric constant *d*_15′_ = 27.6 × 10^−^^12^ C/N and the *max* piezoelectric constant *d*_31′_ = 12.67 × 10^−^^12^ C/N could both be obtained at *θ* = 69° inside three mirror planes. The *max* piezoelectric constant *d*_33′_ = 18 × 10^−^^12^ C/N could be obtained at *θ* = 0°, along the spontaneous polarization axis.

(3) The elastic and piezoelectric parameters of BiFeO_3_ along arbitrary orientations were presented, and by adopting optimal directions these parameters could be significantly enhanced, which shows applications for the growth of BeFeO_3_ films with oriented structures and enhanced properties.

## Figures and Tables

**Table 1:** Comparison of elastic stiffness *c_ij_*_′_ (GPa) and piezoelectric constant *d_ij_*_′_ (10^−12^ C/N) and their extremums along chosen orientations among BiFeO_3_ (*3m*), tetragonal BaTiO_3_ (*4mm*), and KNbO_3_ (*m2m*) [28,29] ^#^.

Crystal||[*nkl*]	[100]	[010]	[001]	Max	Min	Crystal||[*nkl*]	[100]	[010]	[001]	Max	Min
*^BF^c*_11__′_	213	188	213	213	188	*^BT^c*_11__′_ [28]	271	271	271	271	271
*^BF^c*_12__′_	49	49	111	132	44.6	*^BT^c*_12__′_ [28]	152	152	179	179	152
*^BF^c*_13__′_	111	111	49	132	44.6						
*^BF^d*_15__′_	25.9	0	9.8	27.6	0	*^BT^d*_15__′_ [29]	392	392	0	392	0
						*^KN^d*_15__′_ [29]	206	206	21	206	21
*^BF^d*_31__′_	11.9	0	4.5	12.67	0	*^BT^d*_31__′_ [29]	0	0	−34.5	0	−34.5
						*^KN^d*_31__′_ [29]	0	0	19.5	19.5	0
*^BF^d*_33__′_	13	13	18	18	0	*^BT^d*_33__′_ [29]	20	20	85.6	157.8	20
						*^KN^d*_33__′_ [29]		0	29.3	59.3	0

^#^ The symbols BF, BT, and KN represent BiFeO_3_, BaTiO_3_, and KNbO_3_, respectively.
